# Authentic Leadership and Psychological Well-Being of Nurses: A Mediated Moderation Model

**DOI:** 10.1155/2023/7593926

**Published:** 2023-02-27

**Authors:** Stephen Teo, Andrei Lux, David Pick

**Affiliations:** ^1^Northumbria University, Newcastle Upon Tyne, UK; ^2^Edith Cowan University, Perth, Australia; ^3^Curtin University, Perth, Australia

## Abstract

**Aims:**

This study investigates how authentic leadership influences the psychological well-being of Australian nurses. We examined whether authentic leadership could reduce the prevalence of workplace incivility and tested whether shared values and person-organization (P-O) fit could moderate the relationship between workplace incivility and psychological well-being (PWB). A mediated moderation model underpinned by social learning theory was developed to test the influence of authentic leadership on PWB.

**Design:**

We adopted a descriptive correlational research design to test the hypothesized model with a cross-sectional sample of Australian nurses using an online survey. Data were collected across two-waves separated by a six-month interval (*N* = 230, response rate = 38.3%) to minimize the potential effects of common source bias. The hypotheses were tested using Hayes Process Macro (Model 14) on IBM SPSS.

**Results:**

The hypothesized model had good fit indices and supported the mediated moderation model. There was no support for the direct association between authentic leadership and PWB. The supervisor authentic leadership behavior was negatively associated with workplace incivility and PWB. The association between incivility and PWB was positively associated with P-O fit. Nurses with high P-O fit reacted strongly to the positive effect of authentic leadership in reducing workplace incivility, such that they experienced higher levels of PWB.

**Conclusion:**

Authentic leadership behavior is important in the healthcare workplace. It reduces workplace incivility and improves PWB for nurses with high levels of congruence. Implications: our study suggests that senior management should deploy strategies through which frontline supervisors can learn and enact authentic leadership behaviors. They will then be better equipped to improve the PWB of their followers by minimizing the prevalence of workplace incivility. Impact: the study found a significant indirect relationship between authentic leadership behavior and psychological well-being, as mediated by workplace incivility and moderated by person-organization fit. The findings highlight the importance of positive leadership behaviors on the well-being outcomes of nurses in Australia.

## 1. Introduction

Nurses work in a high-stress, high-demand environment that creates considerable job strain [1]. This challenging work context is made even more difficult by mistreatments such as workplace incivility [2], which is a source of psychological stress [3]. Workplace incivility is the result of workplace interpersonal as conflict, evident by rude, disrespectful, and discourteous behaviors that are in violation of mutual respect [4, 5]. Workplace incivility behaviors create situations where nursing and allied staff are distracted from their duties in ways that could compromise the quality of nursing care [6]. Workplace incivility tends to be lower in intensity than most workplace deviant behaviors and has an ambiguous intent to cause harm to others, which makes monitoring and dealing with perpetrators and targets a challenging task [5]. Where it occurs, workplace incivility is disruptive to the work environment because it breaches accepted standards of professional conduct and norms of civility [7]. Such behaviors could result in significant harm to employees, to the organization, and patients [8] and could affect nurses' intention to stay [9]. In this study, we will focus on workplace incivility and the extent to which authentic leadership can help reduce its prevalence and mitigate its negative effect on PWB. The influence of leadership behaviors on followers' well-being is an under-researched topic generally [10] and in the nursing management literature (see [11]).

In this paper, authentic leadership is defined as “a pattern of transparent and ethical leader behavior that encourages openness in sharing information needed to make decisions while accepting input from those who follow” [12], (p. 424). Lemoine et al. [13]; (p. 2051) noted that “authentic leadership is primarily concerned with a leader's self-awareness, self-regulation, and self-concordance, and modeling these characteristics to subordinates….” This positive, relational leadership behavior [14] is particularly relevant in the healthcare setting [15]. Authentic leaders promote healthy work environments to mitigate workplace incivility and, in turn, enhance their followers' well-being [13]. Authentic leaders are self-reflective, listen to feedback, and practice empowerment, which impact positively on nurses' well-being [15]. Nursing leaders who exhibit authentic leadership behavior are grounded in their moral and ethical values.

Nelson et al. [11] note that different ways of operationalizing PWB include a plethora of approaches that range from perceived work stress [16] to exhaustion [2] and psychological distress [17]. We deployed “psychological distress” to conceptualize and measure PWB [18] because PWB is an outcome of the interplay between interpersonal relationships at work [19]. This particular approach to operationalizing “psychological distress” is commonly found in other studies conducted on the work and well-being of nurses [17, 20, 21]. Psychological distress has been used as an indicator of well-being by the World Health Organization [22].

**Figure 1:**
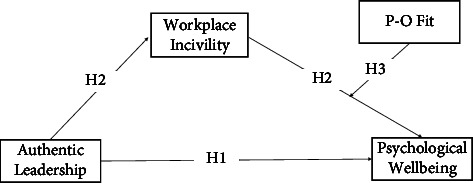
Proposed mediated moderation model.

To the best of our knowledge, the hypothesized indirect relationships have not been empirically tested. The mediated moderation model we propose (see Figure 1) is theoretically informed by social learning theory (SLT) [23, 24]. SLT is used to conceptualize why followers learn from their leader's authentic, moral, and ethical behaviors to minimize workplace incivility. Followers learn what is socially acceptable by observing the values and behavior exhibited by their supervisors. When supervisors display authentic leadership behaviors, those they supervise are likely to adopt similar work values and behave accordingly. P-O fit is a form of subjective value congruence and is defined as the “compatibility between people and the organizations in which they work” [25], p. 1). The alignment of one's own values with their organization's values is critical to employees' work attitudes [26] and stress [27].

In this study, workplace incivility is an example of workplace mistreatment that impacts negatively on the PWB of nurses. It is hypothesized as a mediator, while person-organizational (P-O) fit is hypothesized as a moderating variable of the direct association between authentic leadership and PWB. This study contributes to the literature by unpacking the indirect (mediation and moderation) mechanisms driving the direct and indirect influence of authentic leadership on PWB.

## 2. Background

### 2.1. Direct Relationship: Authentic Leadership and PWB

Nursing is an occupation where employees regularly encounter high levels of psychological stress, which often leads to chronic health problems [1]. Psychological stress is defined as “a particular relationship between the person and the environment that is appraised by the person as taxing or exceeding his or her resources and endangering his or her well-being” [19], p. 19). As an example of PWB [18], psychological distress is an outcome of the interplay between a person and their work environment [19]. Underpinned by positive psychology, authentic leadership has been proposed to be an effective tool for improving the PWB of nursing professionals [28]. Authentic leaders “are guided by sound moral convictions and act in concordance with their deeply held values, even under pressure, and strive to understand how their leadership impacts others” [29], p. 332). Authentic leadership is comprised of four components: self-awareness, internalized moral perspective, balanced processing, and relational transparency [28]. Authentic leadership contributes to a healthy work environment, which is beneficial for staff and patients [28]. Authentic leadership has the potential to build trust and confidence, which in turn results in higher PWB when employees feel valued and respected [11]. Followers' experiences of leader authenticity can be conceptualized as a resource that can be deployed to help improve their well-being at work [16]. We hypothesized that:

H1. There is a positive, direct association between the supervisor's enactment of authentic leadership behaviors and followers' PWB.

### 2.2. Authentic Leadership, Workplace Incivility, and PWB

We theorize that the direct relationship between authentic leadership and PWB is mediated by workplace incivility. To our knowledge (see the systematic review by [28]), there are few empirical studies that examine this mediation relationship. To address this gap in our knowledge, we contend that authentic leadership can help to eliminate workplace incivility, which then improves followers' PWB. While authentic leadership has the potential to reduce the incidence of workplace incivility, our review of existing research brought to light only one study in the context of nursing [2].

Workplace incivility has received considerable research attention over the past three decades (e.g., [5]). Incivility is a “subtype of workplace mistreatment that is characterized by low-intensity social interactions that violate norms of respect and whose harmful intent is ambiguous” [30], p. 316. It occurs far more frequently [4] and can be just as harmful [31] as other forms of harmful behavior. Workplace incivility has a negative association with the PWB of targets [31, 32].

Bandura's [23] SLT can be used to explain the indirect association between authentic leadership and PWB in that while authentic leaders influence the ethical conduct of their followers as a result of role modeling, they also create the work environment by signaling to their team the importance of respect and trust in the workplace [28]. These behaviors form the acceptable standard, which then diffuses across and trickles down the organization via social learning [33], encouraging others to behave in a similar manner. In this way, authentic leaders encourage employees to treat others fairly and with respect while discouraging violations of these expectations [34]. This social learning process can be an influential mechanism through which authentic leaders can affect followers' well-being at work [35] by encouraging civility norms [2]. We hypothesize the following relationship:

H2. Workplace incivility has an indirect mediation effect on the association between the enactment of authentic leadership behaviors and followers' PWB.

### 2.3. P-O Fit as Moderator

As an example of value congruence [36], P-O fit allows “judgments of congruence between an employee's personal values and an organization's culture” [36], p. 875). Avolio and Gardner [37] noted that leaders who exhibit authentic leadership behaviors tend to develop and foster high relational quality and close relationships with their followers, which, in turn, foster greater value congruence (or P-O fit) and follower reciprocation in the behaviors consistent with the leaders' values. Such reciprocity is acquired in the process of social learning and affects followers' well-being (Zheng et al., 2022). Mackey et al. [27] conceptualize P-O fit as a personal resource to buffer workplace stress “because perceptions of organizational fit are generally sought after and valued, which provides stress-resistance potential” (p. 459) as it improves employees' health and well-being. Meta-analytical review findings from Kristoff-Brown et al. [26] support the notion that higher levels of P-O fit are associated with lower levels of work-related stress as employees tend to express a higher level of well-being when they perceive a fit of their own values with their employers. Workplace incivility as an example of stressful work events in the healthcare setting [2, 28] could then be buffered by the extent to which nurses perceived P-O fit. We therefore hypothesize that:

H3. The relationship between workplace incivility and PWB is moderated by P-O fit such that when value congruence is high, the negative effects of workplace incivility on PWB will be weaker.

## 3. Methods

At the time of data collection, there were 352,838 registered nurses and midwives (including registered nurses, enrolled nurses, and midwives), as reported by the Australian Institute of Health and Welfare [38]. PureProfile, a research company based in Australia, was engaged to assist with sending out an online survey to their panel members (inclusion criteria: nurses from Australia, between 18 and 65 years old, full and part-time employment, employment in public, private, and not-for-profit hospitals). This recruitment strategy is common in the literature (e.g., [39]. Our exclusion criteria included those who were not residents of Australia at the time of the study, not qualified as “nurses” as defined by the Australian Health Practitioner Regulation Agency, and those who were older than 65 years old.

We received useable responses from 600 Australian nurses in Wave 1 of data collection. They provided data on demographic and control variables, authentic leadership and P-O fit, and workplace incivility. The respondents were contacted again six months later to provide their responses for their PWB at Time 2. In this cross-sectional study, we obtained 230 useable matched responses (response rate = 38.3%). We decided to use a six-month temporal separation between waves as informed by the incivility literature (see [40]). G∗Power analysis concluded that this sample size has sufficient power and effect size to yield significant accuracy and flexibility of predictions with three predictors [41].

### 3.1. Measures

We adopted previously validated scales in this study. The composite reliability coefficient (CR), average variance estimate (AVE), and maximum shared variance (MSV) are reported below.

#### 3.1.1. Authentic Leadership

We used the 16-item Authentic Leadership Questionnaire (ALQ) from Walumbwa et al. [14] to measure respondents' perceptions of their immediate supervisor's authentic leadership behaviors. It is deemed appropriate to ask employees to provide an assessment of their supervisor's leadership behavior if they have daily interaction with them and can observe whether they demonstrate those behaviors [34]. Sample item includes “Displays emotions exactly in line with feelings.” The items were rated on a five-point Likert type scale, ranging from 1 “not at all” to 5 “frequently, if not always” (CR 0.98, AVE 0.93, and MSV 0.32).

#### 3.1.2. Person-Organization Fit (P-O Fit)

We used the 3-item scale developed by Cable and DeRue [36] to operationalize P-O fit. This scale has been used in the literature [42]. A sample item reads: “My personal values match the organization's values.” These were rated on a seven-point Likert type scale, ranging from 1 “strongly disagree” to 7 “strongly agree” (CR 0.95, AVE 0.83, and MSV 0.32).

#### 3.1.3. Workplace Incivility

We used the 5-item scale from Cortina et al. [31] to measure workplace incivility. A sample item asks respondents if they had experienced various workplace incivility in the previous six months, such as “Addressed you in unprofessional terms, either publicly or privately?” (ranging from 0 “never” to 4 “frequently “at least once a day”)”. The content and discriminant validity of this scale are well established [31]. In this study, we found the validity and reliability coefficients to be satisfactory (CR 0.95, AVE 0.74, and MSV 0.13).

#### 3.1.4. Psychological Well-Being (PWB)

We used the Kessler K-10 Psychological Stress Scale [43] to measure PWB at Time 2. This scale is comprised of 10 commonly found stress symptoms, and in Australia, the K-10 scale has been used as an indicator of well-being in the 2017–18 National Health Survey [44]. It has been used to measure PWB in the nursing profession [17, 20] and in workplace incivility research [45]. Respondents were asked to indicate how frequently they have experienced these stress symptoms over the past 30 days (sample item: “Did you feel that everything was an effort?”). Responses were recorded on a five-point Likert type scale ranging from 1 “none of the time” to 5 “all of the time.” This scale was reverse-coded such that low scores represent distress (low PWB). Validity and reliability coefficients were found to be satisfactory (CR 0.95, AVE 0.64, and MSV 0.13).

#### 3.1.5. Control Variables

In addition to adopting temporal separation in data collection [46], we also controlled for the effects of gender, age, the average number of patients per shift, and organizational tenure because these variables have previously been found to be associated with incivility [47].

### 3.2. Ethical Considerations

Ethics approval was obtained before data collection from Curtin University (reference: SOM 19–12). Consistent with good practice, we assured the participants' anonymity and confidentiality in the participant information letter.

### 3.3. Data Analysis

We used IBM SPSS v.25 to perform bivariate correlations and descriptive statistics. Confirmatory factor analysis was conducted within IBM AMOS v25, and model testing was completed using Model 14 of Hayes' [48] Process Macro within IBM SPSS v27.

### 3.4. Validity and Reliability

All of the scales exceed the recommended reliability thresholds [49]. The Fornell and Larcker's [50] Average Variance Extracted (AVE) test was used to establish the discriminant validity of the scales used in the survey. All of the scales met the 0.50 threshold for AVE, and the square root of the AVE for each scale was higher than its correlation with any other scale. This finding indicates that the scales measured distinct latent constructs.

We used a time-lag research design [46] to minimize common method variance (CMV). As noted by Podsakoff et al. [46], the inclusion of a moderator (incivility × P-O fit) in the model ensures that CMV would not produce statistically significant effects. We also employed proactive procedural remedies such as the random ordering of survey items and ex-post statistical tests (see [46]) to further minimize any potential effects of CMV. To this end, we also conducted Harman's single factor test by subjecting all items to an unrotated exploratory factor analysis which revealed a single largest factor that explained 29.9% of the variance. Given this result, we were confident that CMV effects were not present.

Before testing the 4-factor hypothesized model (that is, authentic leadership, workplace incivility, P-O Fit, and PWB), we conducted several nested measurement model comparisons against three (authentic leadership + workplace incivility, P-O Fit, and PWB), two (authentic leadership + workplace incivility, P-O Fit and + PWB) and single (authentic leadership + workplace incivility + P-O Fit + PWB), factor alternate models. The results indicate that the hypothesized 4-factor model was the best fit to the data (*χ*^2^/d*f* = 1.33, CFI = 0.98, TLI = 0.98, RMSEA = 0.04, SRMR = 0.04) and corresponds with our hypothesized framework.

## 4. Results

As previously reported, 230 Australian nurses completed the two-wave online questionnaire. The majority of the respondents were female (84.3%). Half of the respondents worked part-time (53.5%), and most were employed by public and not-for-profit sector health care organizations (66.1%). Respondents were from New South Wales (33.9%), Victoria (23.0%), Queensland (16.5%), South Australia (12.6%), and Western Australia (9.1%). Over half of the respondents were between 41 and 60 years old (53.9%), followed by those who were 31–40 years old (20.0%), which matches the 2017 data produced by the [51] report (53.6% and 19.6%, respectively) and is consistent with the nationwide health workforce data for nurses and midwives. The largest group of nurses had greater than ten years of organizational tenure (30.4%), followed by those with six to ten years (23.9%), and then those with three to five years (16.1%). The respondents had on average 18.7 patients per shift (SD = 36.7).

Descriptive statistics and zero-order correlations are reported in Table 1. On average, nurses reported their immediate supervisor's leadership behavior to be moderately authentic (*M* = 3.27, SD = 0.87). Ratings of incivility frequency showed 40 nurses had no experience with incivility (17.4%), 78 experienced incivility once every few months or less (34.0%), 51 experienced incivility once a month (22.2%), 46 experienced incivility at least once a week (20.0%), and 15 nurses experienced incivility at least once a day (6.4%). This finding is consistent with the literature, as nurses tend to experience a high level of workplace incivility (see [45, 52]). They also reported a moderate level of value congruence with their organization (*M* = 4.06, SD = 1.55).

To test the hypothesized relationships, we conducted a multiple regression analysis using Model 14 of Hayes's [46] PROCESS macro with 10,000 bootstrapped subsamples using IBM SPSS v25. Control variables were also entered into the model. There was a positive association between workplace incivility and average patients per shift (*β* = 0.17, *p* < 0.05) and a negative association with age (*β* = −0.07, *p* < 0.05). There was a positive association between PWB and gender (*β* = 0.31, *p* < 0.001) and a negative association with supervisory position (*β* = −0.23, *p* < 0.01). There was no support for a direct relationship between authentic leadership and PWB (hypothesis 1), while the remaining hypotheses were supported (see Table 2). There was a negative association between nurses' perception of their supervisor's authentic leadership and workplace incivility (*β* = −0.33, *p* < 0.001). Workplace incivility had a negative association with PWB (*β* = −0.53, *p* < 0.05).

**Figure 2:**
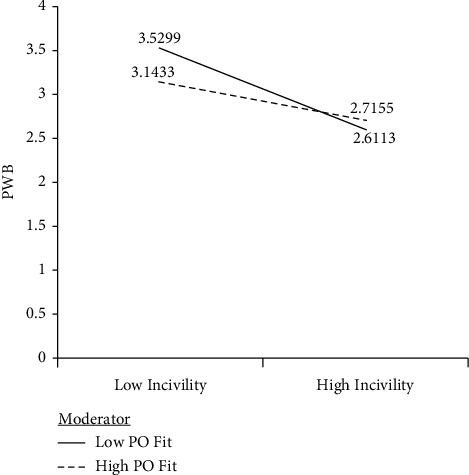
Moderation effect of P-O fit. Note: control variables are not shown in the figure.

Results of the indirect paths are reported in Table 3. Workplace incivility mediated the effect of authentic leadership on PWB (effect −0.13, SE 0.05, 95% CI [−0.24, −0.03]), and there was support for P-O fit to moderate the full mediation of authentic leadership on PWB (effect −0.048, SE 0.017, 95% CI [−0.084, −0.016]). Hypothesis 2 was supported. Nurses' P-O fit moderated the negative relationship between workplace incivility and PWB (*β* = 0.16, *p* < 0.01). The moderation plot (see Figure 2) indicates that P-O fit minimized the negative association between workplace incivility and PWB. As shown in Table 3, these results provide evidence to support a mediated moderation model.

## 5. Discussion

The main aim of this study was to respond to the call in the literature to investigate the impact of leadership behaviors on the well-being of followers. As part of this research, we tested the mediator effect of workplace incivility and value congruence (measured by P-O fit) as a moderator. Our findings have three main implications.

First, our study contributes to the literature by enhancing our understanding of authentic leadership [2, 28] in PWB, especially in minimizing psychological distress. This finding contributes to the underresearched topic of how leadership behaviors affect followers' well-being [10, 11]. By establishing an indirect association between authentic leadership and PWB, our study has provided additional evidence of the effect of authentic leadership in minimizing workplace incivility, and in doing so, sheds light on how authentic leadership influences the PWB of nurses at different stages of their work tenure beyond early career and initial job experiences [2, 53].

The application of social learning theory [23] allows us to unpack how this process takes place in the nursing context [24]. Supervisors who engage in authentic leadership behaviors are likely to discourage workplace incivility by openly displaying and upholding ethical norms and values regarding acceptable workplace conduct [2]. Followers learn that this is the accepted social norm and imitate their leader's positive behaviors. This in turn reduces the prevalence of workplace incivility and improves PWB. Authentic leadership behaviors enable nurse managers to create a more civil work environment and enhance employees' well-being at work (e.g., [35]).

Second, we found workplace incivility (a form of workplace deviance) had a negative association with nurses' PWB. While there are a variety of approaches to operationalizing and measuring PWB evident in previous research, in this study we have demonstrated how psychological distress as an indicator for PWB [18] could be enhanced by minimizing interpersonal deviance at work. Workplace incivility depletes the psychological resources of those who are affected by the behaviors [27]. Employees could learn positive leadership behaviors by mirroring the authentic behaviors of their supervisors, which in turn helps to minimize the prevalence of workplace mistreatment across the organization [54].

Third, P-O fit was found to be a moderator of the association between workplace incivility and PWB. As indicated by the moderation plot, the moderation effect occurs when the P-O fit is high. What we have is a situation where the enactment of authentic leadership behaviors leads to less workplace incivility. This leads to a higher level of PWB for nurses who exhibit a high level of P-O fit. P-O fit, as a form of value congruence, could be treated as a resource that nurses could draw upon to maximize the positive consequences of civility. This moderation finding represents a contribution to the literature as it has not been empirically tested [27]. Our finding also adds to our understanding of the contribution of P-O fit on employee well-being outcomes at work [26].

### 5.1. Practical Implications

This study has implications for nurse managers as well as managers in the health and public sectors generally. Our findings suggest that health care managers should be trained to develop their positive, relational leadership styles. The enactment of authentic leadership behaviors by frontline supervisors combats workplace stressors and improves well-being by minimizing workplace incivility in healthcare settings. Authentic leadership could lead to the development of more civil [2], heathy, and safe workplaces that minimize employee mistreatment [54]. This influence is reinforced through an improvement in the P-O fit between nurses' personal values and those of the organization where they work. As a form of ethical leadership behavior, authentic leadership has the potential to significantly reduce workplace incivility and increase PWB among employees in high-demand work environments like nursing.

Workplace incivility could be minimized by introducing workplace civility programs, which could be used to reduce incivility in order to improve job attitudes and well-being [2, 55]. An example is the 6-month workplace intervention model known as CREW - Civility, Respect, and Engagement at Work [56]. As part of the intervention, nurses would meet with their coworkers within their workplace on a “ weekly or biweekly basis to work on effective interpersonal interactions at work” [56]. Trained facilitators would provide guidance to the groups on how to improve workplace communication. This intervention seeks to improve workplace social relationships in order to enhance respect. A civility toolkit could also be produced, similar to the one proposed by the UK's National Health Service (NHS). The NHS used the toolkit to create a civil and respectful culture to improve employee well-being and patient care [52]. These organizational practices are important as they could improve nurses' person-organization fit and are likely to reciprocate with affective commitment and a greater sense of belongingness. Nursing supervisors who exhibit authentic leadership behavior in the workplace by striving to build open, genuine relationships and by helping their followers fit in with their workplace create more civil work environments that promote employees' well-being and, ultimately improve the quality of care [6].

### 5.2. Limitations and Future Research Implications

Focusing on nurses in the Australian health care sector limits the generalizability of our findings. We applied procedural and statistical remedies to provide assurance that common method bias did not affect our results [46]. Control variables were incorporated to control for confounding effects. We should note the potential for reverse causality remains. Future studies should test the possibility of a reverse causal relationship between authentic leadership and PWB by collecting longitudinal data. Multisource data could be used to develop our findings by better isolating the predictors of PWB. It might also be valuable to explore further the extent to which and how authentic leadership is being measured by using experimental design or implicit measures, or to explore the relationship between leaders' intentions and followers' perceptions of authentic leadership behavior [57]. Recall bias could be another potential limitation, which can be addressed by using a daily diary design [58].

## 6. Conclusion

Our study aimed to examine how authentic leadership behavior affects the well-being of nurses where workplace incivility is present. Using data collected from a sample of nurses working in Australian health care organizations, we found evidence to support the argument that workplace incivility has a deleterious effect on PWB. Our study suggests that nursing managers have an important role to play in protecting and improving the well-being of those they supervise, and authentic leadership behavior is an additional and effective skill in this endeavor [59–63].

## Figures and Tables

**Table 1:** Descriptive statistics and intercorrelations.

	Mean	SD	1	2	3	4
1. Authentic leadership (T1)	3.27	0.87	**0.98**			
2. Workplace incivility (T1)	1.14	1.03	−0.40^∗∗∗^	**0.95**		
3. P-O fit (T1)	4.05	1.54	0.53^∗∗∗^	−0.34^∗∗∗^	**0.95**	
4. Psy well-being (T2) (Low scores in psychological well-being scale represent distress)	2.06	0.94	0.09	−0.49^∗∗∗^	0.07	**0.95**

*Note*. *N* = 230. ^∗∗∗^*p* < .001. Correlations of control variables are not reported. Bold, italicized text: Composite Reliability Coefficient. T1: time 1. T2: time 2.

**Table 2:** Results of mediated moderation analysis using Hayes process macro model 14.

	Unstandardized *β*	SE	*T*	*p*	LLCI	ULCI
DV = workplace incivility (*R*-sq = 0.2522)						
Constant	−0.0498	0.1049	−0.4744	0.6357	−0.2565	0.1569
Gender	−0.0650	0.0636	−1.0225	0.3077	−0.1904	0.0603
Employment status	0.0004	0.0011	0.3842	0.7012	−0.0017	0.0025
Average patients per shift	0.1725	0.0854	2.0199	0.0446	0.0042	0.3408
Age	−0.0687	0.0283	−2.4298	0.0159	−0.1245	−0.0130
Authentic leadership T1	−0.3247	0.0425	−7.6362	0.0000	−0.4085	−0.2409

DV = PWB (*R*-sq = 0.1208)						
Constant	2.5356	0.3226	7.8599	0.0000	1.8998	3.1714
Gender	0.3064	0.1011	3.0318	0.0027	0.1072	0.5055
Employment status	0.0279	0.0621	0.4487	0.6541	−0.0945	0.1502
Average patients per shift	−0.0005	0.0010	−0.4706	0.6384	−0.0025	0.0015
Supervisory position	−0.2252	0.0829	−2.7170	0.0071	−0.3885	−0.0618
Age	−0.0340	0.0285	−1.1932	0.2341	−0.0901	0.0221
Authentic leadership T1	0.0273	0.0531	0.5153	0.6069	−0.0772	0.1319
Workplace incivility T1	−0.3366	0.1419	−2.3714	0.0186	−0.6163	−0.0569
P-O fit T1	−0.0706	0.0420	−1.6834	0.0937	−0.1533	0.0121
Wk incv* × *P-O fit (interaction)	0.1227	0.0384	3.1943	0.0016	0.0470	0.1984

*N* = 230.

**Table 3:** Results of mediated moderation analysis.

Direct effect of authentic leadership on PWB (hypothesis 1)					
Effect	se	*t*	*p*	LLCI	ULCI
0.0273	0.0531	0.5153	0.6069	−0.0772	0.1319

Conditional indirect effects of *X* on *Y*					

Indirect effect: authentic leadership→workplace incivility→PWB (hypothesis 2)					

P-O fit (moderator)		Effect	BootSE	BootLLCI	BootULCI

2.2765		0.0186	0.0263	−0.0319	0.0717
3.6357		−0.0356	0.0222	−0.0807	0.0071
4.995		−0.0897	0.0314	−0.1541	−0.0300

Index of mediated moderation (hypothesis 3)					

		Index	BootSE	BootLLCI	BootULCI

P-O fit (moderator)		−0.0398	0.0137	−0.0680	−0.0140

## Data Availability

The survey data used to support the findings of this study have not been made available because of restriction imposed by institutional review board for sharing data. This was part of the ethics approval conditions.
